# Ciliary body myxoid epithelioid sarcoma in a cat: a case report

**DOI:** 10.1186/s12917-024-04286-3

**Published:** 2024-10-01

**Authors:** Marina L Leis, Soraya Sayi, Bruce H Grahn

**Affiliations:** Western College of Veterinary Medicine, University of Saskatchewan, Saskatoon, SK Canada

**Keywords:** Myxoid sarcoma, Ciliary body tumor, Cat

## Abstract

**Background:**

The majority of primary, intraocular tumors in cats originate from the uvea and include feline diffuse iris melanoma, lymphoma, and iridociliary epithelial adenoma or adenocarcinoma. In this case report, we describe for the first time the clinical, histological, and immunohistochemical findings of a rare myxoid intraocular neoplasm arising from the ciliary body in a cat.

**Case presentation:**

A 14-year-old, female, spayed domestic shorthaired cat was presented for evaluation of discolouration of the right eye. Upon examination, a clear to light whitish-tan, bubble-shaped intraocular mass adherent to the inferior ciliary body and extending into the anterior chamber was noted. Within five weeks, the tumor was significantly larger and the eye had developed secondary glaucoma so was enucleated. Light microscopic examination of the globe revealed a multinodular, hypocellular neoplasm arising from the ciliary body composed of interwoven spindle cells embedded in abundant amounts of a lightly basophilic myxoid matrix. Neoplastic cells exhibited strong immunoreactivity for cytokeratin while also showing moderate to strong immunoreactivity to vimentin. A diagnosis was therefore made of an unusual intraocular myxoid epithelioid sarcoma arising from the ciliary body.

**Conclusions:**

Although apparently exceedingly rare, epithelioid myxosarcoma should be included as a differential diagnosis for intraocular tumors in cats and they represent a clinical, histologic, and immunohistochemical diagnostic challenge. Early surgical intervention should be considered to prevent local invasion and ascension to the brain.

## Background

The majority of intraocular tumors in cats originate from the uvea. Melanocytic tumors (feline diffuse iris melanoma or FDIM) are the most common uveal tumor in cats [1]. FDIM is associated with a large degree of variation in cellular morphology. They do not present as a discrete mass but rather diffuse tumor growth invades the superficial iris first and the deeper iris later, and over time may invade the iridocorneal angle and trabecular meshwork.

Uveal lymphoma is the second most common intraocular tumor of cats and most commonly involves the anterior uvea. Though uveal lymphoma is most often associated with multicentric disease, solitary ocular lymphoma in cats has been reported [2].

Feline post-traumatic ocular sarcoma (FPTOS) has both spindle and round cell variants. It arises from malignant transformation of lens epithelial cells following trauma to the lens and chronic uveitis. Evidence of a lens rupture is required for diagnosis and there is an average of 7 years between intraocular trauma and diagnosis of FPTOS [3].

Tumors of neuroectodermal origin are seen less frequently [1]. Most are iridociliary adenomas or well-differentiated iridociliary adenocarcinomas arising from the neuroectoderm of the ciliary body or posterior iris. They often grow in chains and cords around the lens equator and invade the anterior chamber. Medulloepitheliomas fall within the category of primitive neuroectodermal tumors (PNET) and are very rare in the cat [4].

In this report, we describe the clinical, histological, and immunohistochemical findings of an unusual myxoid intraocular neoplasm arising from the ciliary body in a domestic shorthaired cat.

## Case presentation

**Fig. 1:**
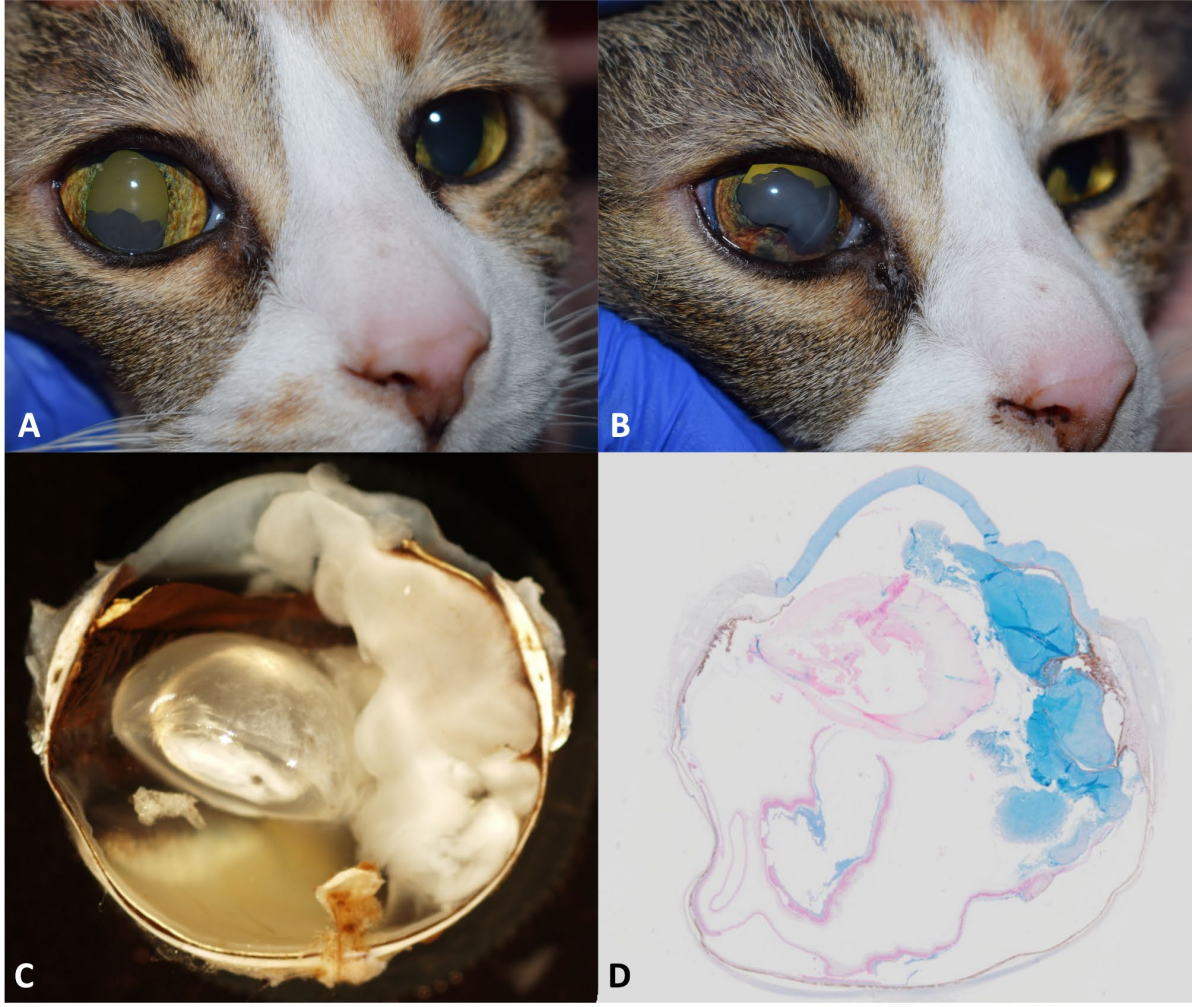
(**A**) Retro-illuminated clinical photograph at initial presentation demonstrating a tan intraocular mass adhered to the inferior ciliary body (**B**) Five weeks following initial presentation, rapid growth of the mass, invasion of the iris, and dyscoria were noted (**C**) Gross section photograph of the eye showing a white gelatinous mass extending from the ciliary body, into the posterior segment, through the pupil, and into the anterior chamber (**D**) Histologic section of the eye stained with alcian blue demonstrating the presence of mucopolysaccharides in the myxoid matrix

A 14-year-old, female, spayed domestic shorthaired cat was presented for evaluation of a right ocular lesion. The owner had noticed discolouration of the eye of 3 months duration. Examination by a veterinary ophthalmologist revealed normal menace responses, pupillary light reflexes, and oculocephalic reflexes. Schirmer tear testing was 23 and 22 mm/min in the right and left eye, respectively, and intraocular pressure was estimated at 22 and 16 mmHg in the right and left eye, respectively. Biomicroscopic slit lamp examination of the right eye revealed a white to tan intraocular mass adhered to the inferior ciliary body and protruding into the anterior chamber through the pupil (Fig. 1A). The remainder of the anterior segment examination of the right eye was within normal limits and indirect ophthalmoscopy was unremarkable. There were no abnormalities on biomicroscopic and indirect ophthalmoscopic examination of the left eye. At this time the list of differentials included a ciliary body adenoma, adenocarcinoma, or medulloepithelioma, and a recommendation for enucleation was made. Five weeks later, the cat was represented to the ophthalmologist who noted a significant enlargement of the intraocular mass, a reduction in pupillary movement and dyscoria due to local invasion of the mass, and an intraocular pressure of 35 mmHg confirming secondary glaucoma (Fig. 1B).

**Fig. 2:**
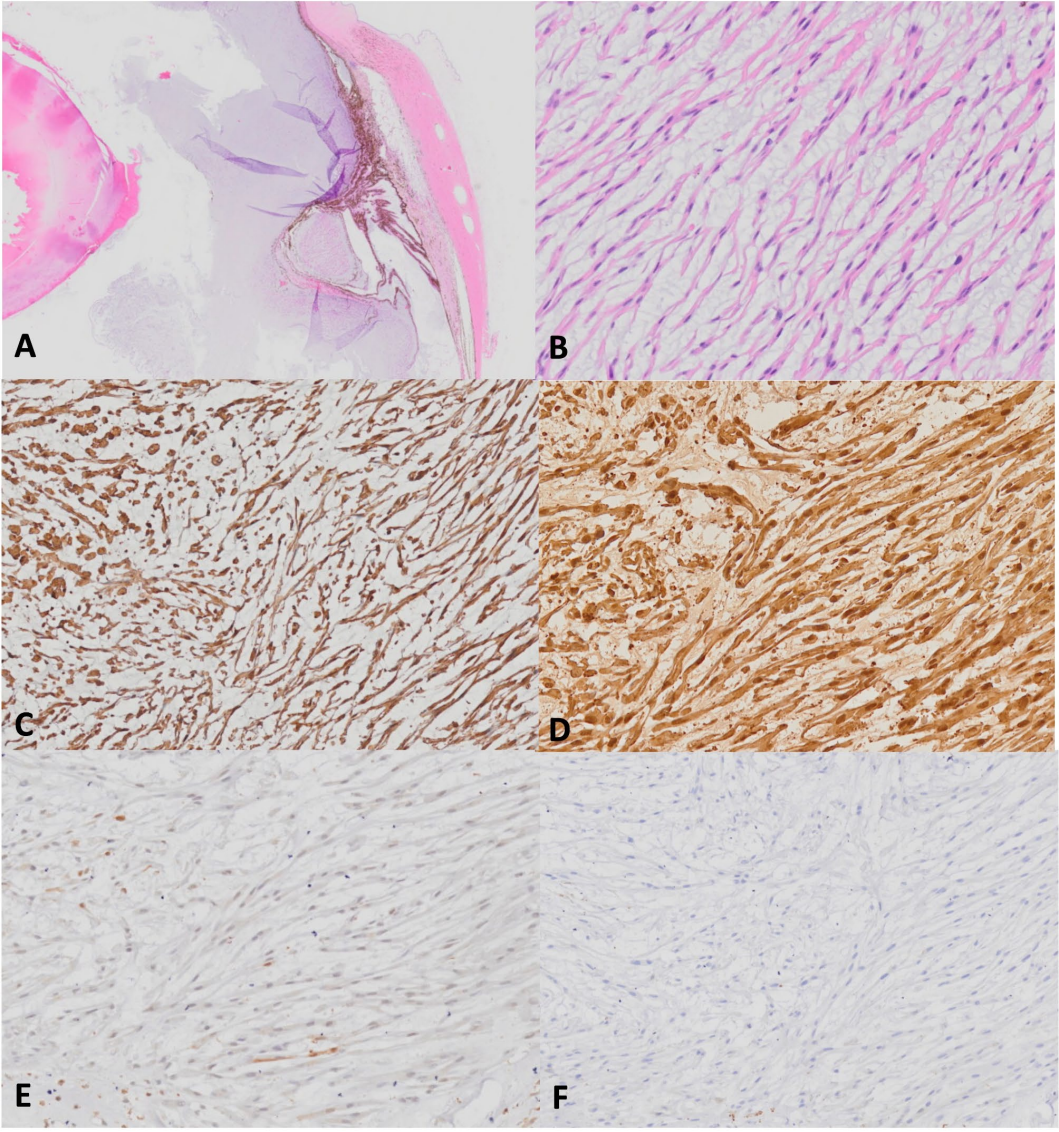
(**A-B**) Hematoxylin and eosin sections demonstrating the neoplasm composed of interwoven spindle cells embedded in abundant amounts of a lightly basophilic myxoid matrix. (**C-F**) Immunohistochemical stains showing strong tumor immunoreactivity for cytokeratin (**C**) and vimentin (**D**) and weak immunoreactivity for S100 (**E**) and actin (**F**)

A right-sided, subconjunctival enucleation was performed and the globe was fixed in 10% neutral-buffered formalin and submitted to the Prairie Ocular Pathology Service in Saskatoon, Saskatchewan, for histologic evaluation. Gross examination of the eye revealed a white gelatinous mass that extended from the ciliary body, through the pupil and into the anterior chamber and posterior segment (Fig. 1C). Sections were routinely processed with haematoxylin and eosin (H&E) and Alcian blue (Fig. 1D). Microscopic examinations revealed a multinodular, hypocellular neoplasm arose from the non-pigmented ciliary epithelium and extended into the posterior segment and into the anterior chamber (Fig. 2A-B). The neoplasm was composed of interwoven spindle cells embedded in abundant amounts of a lightly basophilic myxoid matrix. Occasional cells were round to polygonal. The cells had pale eosinophilic cytoplasm with discrete cell borders and ovoid to elongated nuclei with inconspicuous nucleoli. Mitotic figures were uncommon (< 1 per 10 high power fields). A few binucleated and multinucleated forms were present. The myxoid change was extensive and accounted for over 50% of the tumor mass.

Differential diagnoses considered for primary, feline intraocular neoplasia included feline diffuse iris melanoma (FDIM spindle-cell variant), ciliary adenocarcinoma, and feline post-traumatic sarcoma (FPTS) and were excluded based on histologic features, pattern of distribution of cells, and immunohistochemical staining pattern. Other differentials considered at this point included a myxosarcoma, a myxoid variant of a nerve sheath tumor, a myxoid leiomyosarcoma, or a myofibroblastic tumor.

Immunohistochemistry was performed to identify the cell of origin using a panel of markers: actin, desmin, factor VIII-related antigen, cytokeratin, S-100, cytokeratin, and vimentin (Table 1). Neoplastic cells exhibited strong immunoreactivity for cytokeratin and showed moderate to strong immunoreactivity to vimentin (Fig. 2C-D). A small percentage of cells had weak to moderate intensity to S-100 and some showed weak staining for smooth muscle actin (Fig. 2E-F). The neoplastic cells were negative for desmin and factor VIII-related antigen.

**Table 1:** Immunohistochemistry results for the intraocular neoplasm

Immunohistochemical target	Result
Actin, Muscle-Specific	Negative (< 5% of cells stained with weak intensity)
Desmin intermediate filaments	Negative
Factor VIII Related Antigen	Negative
Keratin intermediate filaments	Positive (> 99% of cells stained with moderate to strong intensity)
S-100 protein	Negative (< 5% of cells stained with weak to moderate intensity)
Vimentin intermediate filaments	Positive (> 95% of cells stained with moderate to strong intensity)

Based on the cellular morphology, histochemical and immunohistochemical characteristics of the neoplasm, a diagnosis was made of a primary, intraocular myxoid epithelioid sarcoma arising from the ciliary body. Ocular metastasis from a primary systemic site was deemed unlikely based on the lack of diffuse uveal involvement, intravascular presence within the uveal blood supply, and a lack of carpeting of the uvea that are most typical of metastatic carcinomas.

## Discussion and conclusions

We describe for the first time the clinical, histological, and immunohistochemical findings of a rare, primary intraocular myxoid epithelioid sarcoma arising from the ciliary body in a cat. Cytokeratin is a well-established marker for the detection and classification of tumors of epithelial origin (carcinomas) while vimentin is a marker for mesenchymal cell tumors. It was therefore unexpected that the tumor in the case herein had strong co-expression of both keratin and vimentin. Isolated or scattered keratin-positive cells occur relatively frequently in undifferentiated pleomorphic sarcomas [5]. However, in the case presented herein the keratin-positive cells were found diffusely throughout the mass. This may be similar to epithelioid sarcomas in humans, reported to have strong immunoreactivity for keratin and vimentin [5, 6]. Myxoid epithelioid sarcomas are exceedingly rare in humans and represent a diagnostic challenge [7]. According to the authors of one study, the major differential diagnosis is a myoepithelioma which can be ruled out with negative immunoreactivity to S-100^7^.

A single case of feline intraocular myxoid leiomyosarcoma has been reported and unlike the case herein was positive for smooth muscle actin, S100, and vimentin [8]. Similar to the case herein, the mass appeared to be locally invasive and in addition invaded the optic nerve. No recurrence or evidence of metastasis was noted at 6 months post-enucleation. Another case report of intraocular myxosarcoma in a dog revealed extension of the mass into the retrobulbar space, optic chiasm, and brain [9]. These reports and the rapidity at which the mass appeared to be growing over a 5-week period in the case herein raised concern for both local invasion and potential metastasis. Though advanced imaging results to rule out distant metastasis were not available in this case, there was no evidence to support invasion of the angular aqueous plexus, scleral venous plexus, or optic nerve.

Here we describe the first, unusual case of a myxoid epithelioid sarcoma in the eye of a cat. This is also the second report of an intraocular myxoid neoplasm in the cat. Although apparently exceedingly rare, myxoid epithelioid sarcoma should be included as a differential diagnosis for intraocular tumors in cats arising from the ciliary body, and it represents a clinical, histologic, and immunohistochemical diagnostic challenge. Early surgical intervention is recommended to prevent local invasion and ascension to the brain via the optic nerve.

## Data Availability

All data generated during this study are included in this published article.

## Abbreviations

FDIM: Feline diffuse iris melanoma
FPTOS: Feline post–traumatic ocular sarcoma
PNET: Primitive neuroectodermal tumor
